# The Natural Killer Cell Landscape in the Natural History of Hantavirus Cardiopulmonary Syndrome in a Chilean Cohort

**DOI:** 10.3390/v18070712

**Published:** 2026-06-27

**Authors:** Juan Hormazabal, Natalia González, Fernanda Silva-Fuentes, Cecilia Poli, Analía Cuiza, Nicole Le Corre, Marcela Ferres, René López, Jerónimo Graf, Maria Luisa Rioseco, Francisco Arancibia, Ricardo Fritz, Jose Luis Perez, Leonila Ferreira, Mario Calvo, Pablo Vial, Cecilia Vial

**Affiliations:** 1 Programa Hantavirus & Zoonosis, Instituto de Ciencias e Innovación en Medicina (ICIM), Facultad de Medicina, Clínica Alemana Universidad del Desarrollo, Av. La Plaza 680, Las Condes, Santiago 7500000, Chile; jhormazabal@udd.cl (J.H.); fernanda.csilvafuentes@gmail.com (F.S.-F.); lia.cuiza@gmail.com (A.C.); pvial@udd.cl (P.V.); 2 Programa de Inmunogenética e Inmunología Traslacional, Instituto de Ciencias e Innovación en Medicina, Facultad de Medicina, Clínica Alemana Universidad del Desarrollo, Santiago 7610658, Chile; nmgonzalez@udd.cl (N.G.); cpoli@udd.cl (C.P.); 3 Departamento Pediatría Clínica Alemana de Santiago, Av. Vitacura 5951, Vitacura, Santiago 7500000, Chile; 4 Departamento de Enfermedades Infecciosas e Inmunología Pediátricas, Pontificia Universidad Católica de Chile, Marcoleta 391, Región Metropolitana, Santiago 7500000, Chile; n.lecorre.p@gmail.com (N.L.C.); mferres@uc.cl (M.F.); 5 Departamento de Paciente Crítico, Clínica, Alemana de Santiago Av. Vitacura 5951, Vitacura, Santiago 7500000, Chile; rene.lopez@udd.cl (R.L.); jgraf@alemana.cl (J.G.); 6 Grupo Intensivo, Instituto de Ciencias e Innovación en Medicina (ICIM), Facultad de Medicina, Clínica Alemana Universidad del Desarrollo, Av. La Plaza 680, Las Condes, Santiago 7500000, Chile; 7 Regional de Puerto Montt, Universidad San Sebastián, Sede Patagonia, Puerto Montt 5501842, Chile; malurioseco@gmail.com; 8 Pulmonology Department, Instituto Nacional del Tórax, Universidad de Chile, Santiago 8330015, Chile; farancibia@torax.cl (F.A.); rafritzg@gmail.com (R.F.); 9 Institute of Clinical Sciences, Universidad Austral de Chile, Valdivia 5090000, Chile; joseluisperez@uach.cl; 10 Hospital Regional Guillermo Grant Benavente, Concepción 4070038, Chile; lferreira@ssconcepcion.cl; 11 Hospital Regional de Valdivia, Valdivia, Chile; mariocalvoarellano@gmail.com

**Keywords:** *Orthohantavirus andesense*, Andes virus, HCPS severity, NK cell phenotype, CD56dim, innate immunity, NK cell dysfunction, viral immunology, flow cytometry, disease severity

## Abstract

Hantavirus cardiopulmonary syndrome (HCPS) caused by Andes Orthohantavirus (ANDV) carries case-fatality rates up to 40%; however, the innate immune determinants of disease severity remain poorly defined. Natural killer (NK) cells are central mediators of early antiviral immunity, but their landscape during the earliest phase of ANDV infection has not been characterized. Using multiparameter flow cytometry and unsupervised UMAP-based clustering in PBMCs from 13 HCPS patients stratified by severity and nine healthy donors, we show that severe HCPS is characterized by a coordinated disruption of the CD56dim NK cell compartment, encompassing reduced subset frequencies, specific reduction in the terminally differentiated NKG2C+CD57+ adaptive-like pool, and intrinsic impairment of IFN-γ production and degranulation, deficits that were absent in mild patients and persisted in part beyond clinical recovery. Furthermore, CD56dimCD16+ NK cell frequencies correlated negatively with viral load across all acute patients, independent of clinical severity. These findings establish severe HCPS not merely as a state of NK cell depletion, but as one of selective functional impairment of the most cytotoxically competent NK cell population during the critical early acute phase of ANDV infection.

## 1. Introduction

Hantaviruses (Order Bunyaviral, Orthohantavirus, family Hantaviridae) are zoonotic single-stranded, negative-sense RNA viruses, transmitted to humans through inhalation of aerosolized excreta from infected rodents, the primary natural reservoir host of hantaviruses [1,2,3]. These hantaviruses can cause two distinct syndromes: hemorrhagic fever with renal syndrome (HFRS) across Europe and Asia, and hantavirus cardiopulmonary syndrome (HCPS) in the Americas [4,5], both reaching case-fatality rates up to 10% and 40%, respectively [5,6,7].

Hantavirus viral particles are enveloped by a lipid layer ranging from 80 to 120 nm in diameter and harbor a trisegmented negative-sense single-stranded RNA genome. These are called small (S), medium (M), and large (L) segments, which encode the nucleocapsid protein (N), the glycoprotein precursors Gn and Gc, and the RNA-dependent RNA polymerase (RdRp), respectively [1,4]. These structural proteins are essential for viral biogenesis, replication, and assembly, with the Gn/Gc spike complex mediating host cell entry and determining host specificity, representing the most genetically diverse components across hantavirus species.

Among the Americas hantaviruses, the *Orthohantavirus andesense* (ANDV) is the only species where human-to-human transmission has been described, making it a potential pandemic threat and a major public health concern. ANDV is the leading cause of HCPS in Chile and southern Argentina, with the first cases identified in 1995 [5]. In Chile, all confirmed cases are caused by ANDV, with an annual rate of 40–60 cases, a case-fatality rate between ~30–40%, and no approved antiviral therapies nor vaccines [5,8,9].

When a person is infected, HCPS evolves through four phases: incubation (5–42 days, asymptomatic), prodromal (3–6 days, non-specific flu-like symptoms), cardiopulmonary (5–9 days, cardiopulmonary symptoms), and convalescence [10,11,12]. The cardiopulmonary phase is defined by sudden hypoxemia progressing to acute respiratory distress, cardiogenic shock, and hemodynamic collapse. Severe cases require mechanical ventilation (MV) and vasoactive drugs (VD) and often salvage measures as extracorporeal membrane oxygenation. The fatality among ventilated patients reaches 77%, with 90% of deaths occurring within the first 48 h [10,13]. Clinically, not all infected individuals develop this severe clinical course, with a small percentage of patients presenting with mild disease that may only require supplemental oxygen.

In humans, pulmonary vascular endothelial cells are the primary sites of ANDV replication. Infection triggers their cellular reprogramming disrupting tight junctions, and causing capillary leakage that develops to pulmonary edema [4]. Elevated secretion of pro-inflammatory cytokines and chemokines, including CXCL10 and RANTES, amplifies the host immune response against infected endothelium, with serum cytokine levels differing markedly between fatal and non-fatal outcomes [14,15,16]. Notably, no cytopathic effect is observed in infected tissues, indicating that immune-driven host responses rather than direct viral damage underlie HCPS pathogenesis [4].

In viral infections in general, including hantavirus, type I interferon is among the first antiviral pathways to respond, yet both insufficient and excessive activation is associated with poor clinical outcomes, including hantavirus infection [17,18,19]. The precise mechanisms driving HCPS severity remain incompletely defined. However, implicated processes include CD8+ T cell cytotoxicity, TNF-α and Il-6-driven cytokine storm, viral load, platelet dysfunction, and NK cell activation [15,20,21,22,23,24,25]. Among these immune effectors, NK cells have emerged as particularly relevant in viral infections; they can be activated by virus-induced cytokines, (e.g., type I interferons [IFN-α/β]), and their functions are shaped by epigenetic and transcriptional reprogramming during infection [26]. Meanwhile, individuals with inborn NK cell deficiencies exhibit impaired viral clearance and increased disease severity, highlighting their essential role in early antiviral defense [27].

NK cell responses during hantavirus infection have been best characterized in Puumala (PUUV)-driven HFRS, where CD56dim NK cells display robust activation recorded 5 days after onset of the symptoms, with elevated CD69, meanwhile in an experimental in vitro setting, following a haantan (HNTV) infection, there is an increase in the intracellular granzyme B and perforin, driven by hantavirus-induced IL-15/IL-15Rα expression on infected endothelial cells [21,28]. Elevated serum IL-15 correlates independently with disease severity and fatal outcome, and activated NK cells acquire sufficient cytotoxic capacity to kill uninfected endothelial cells despite normal HLA class I expression, suggesting a mechanism that may directly contribute to the vascular leak characteristic of hantavirus disease [16,21]. However, the extent to which these dynamics operate during the earliest phase of ANDV infection and how the NK cell compartment is shaped in the context of HCPS remain poorly understood. We hypothesized that severe HCPS is associated with distinct alterations in NK cell phenotype, maturation, activation status, and/or effector function compared with mild disease, and that these alterations contribute to the immunological mechanisms underlying disease severity. To address this hypothesis, we performed a comprehensive characterization of NK cell subsets, activation markers, and cytotoxic function in acute and convalescent samples from Chilean HCPS patients stratified according to disease severity. Through this approach, we sought to define NK cell contribution to ANDV immunopathogenesis and identify immunological signatures associated with different clinical outcomes.

## 2. Materials and Methods

### 2.1. Study Design and Patient Recruitment

This study was approved by the ethics committee of Clínica Alemana Universidad del Desarrollo (UDD), IRB4858, FWA8639 (approval date: 17 March 2016). All enrolled subjects provided written informed consent, or it was provided by their parents or legal guardians. Clinical data and stored samples were anonymized to ensure confidentiality.

The subjects were recruited from research centers in the central and southern regions of Chile, and hospitalized individuals with suspected or confirmed ANDV infection were invited to participate. Inclusion criteria were A) confirmed hantavirus diagnosis: positive IgM or RT-qPCR for hantavirus in blood plus acute febrile illness of fewer than 12 days; B) presumptive hantavirus diagnosis, requiring all of the following within fewer than 12 days of disease onset: (1) fever, (2) headache, myalgia, nausea, vomiting, diarrhea, or abdominal pain, (3) platelet count <150 × 10^3^/mL, and (4) hypoxia. Patients requiring ECMO at the time of the first sample were excluded. Severe HCPS was defined by the development of cardiopulmonary shock requiring vasoactive drugs (VD) and mechanical ventilation (MV), whereas mild clinical course was defined by prodromal symptoms or minor cardiorespiratory compromise without requirement of MV or VD, with stable hemodynamic parameters. For healthy donors, additional exclusion criteria included pregnancy, chronic disease, and acute infectious symptoms within two weeks prior to enrollment, with all donors confirmed seronegative for hantavirus post-enrollment. Blood samples were collected during the early acute phase (days 0–2 after cardiopulmonary symptom onset) and the convalescent phase (≥60 days); a single sample was taken from healthy donors.

### 2.2. Sample Collection and PBMC Isolation

Peripheral blood was collected in 7.5 mL Vacutainer™ ACD Solution A tubes (BD Biosciences, Milpitas, CA, USA). Briefly, plasma was separated by centrifugation at 1300× *g* for 10 min at room temperature, aliquoted (0.5–0.75 mL), and stored at −80 °C. PBMCs were isolated by density gradient centrifugation using Ficoll-Paque Plus™ in SepMate™-50 tubes at 350× *g* for 20 min at 18–20 °C, washed three times with PBS/2% FBS, resuspended in freezing medium Cryostore at 4–5 × 10^6^ cells/mL, and stored at −80 °C using isopropanol-controlled rate freezing [29].

### 2.3. Flow Cytometry

Cryopreserved PBMCs (~5 × 10^6^ cells) were thawed at 37 °C, washed in RPMI 1640/10% FBS/1% Penicillin-Streptomycin, and centrifuged at 1800 rpm for 10 min prior to staining. All the samples were acquired on a CytoFLEX cytometer (Beckman Coulter, Brea, CA, USA). Two panels were used:

Panel 1—Functional Characterization: NK cell degranulation was assessed under stimulated and unstimulated conditions. For stimulation, ~1 × 10^6^ PBMCs were co-incubated with K562 tumor cells (ATCC) at a 1:25 ratio (40,000 K562 cells per 10^6^ PBMCs) in 200 µL RPMI/10% FBS/1% P/S for 5 h at 37 °C with 5% CO_2_. Anti-CD107a-APC (BioLegend) was added at the start of incubation. After 1 h, Brefeldin A and GolgiStop (both BD Biosciences, 1000×) were added to block intracellular trafficking for the remaining 4 h. The cells were then stained with LIVE/DEAD Fixable Near-IR (Thermo Fisher Scientific, Cambridge, MA, USA) for viability, followed by surface staining with anti-CD16-V500 and anti-CD56-PE-Cy7 (BD Biosciences). Intracellular staining was performed after fixation and permeabilization with the BD Cytofix/Cytoperm kit, using anti-Perforin-BV421, anti-CD3-BUV496, and anti-IFN-γ-Alexa Fluor 700 (BioLegend, San Diego, CA, USA); CD3 was stained intracellularly given its internalization upon cellular stimulation, ensuring reliable T cell exclusion from the NK cell gate. The samples were fixed with BD Stabilizing Fixative until acquisition. Of note, these markers were selected to capture distinct aspects of NK cell effector function: CD107a as a degranulation marker, IFN-γ as a measure of cytokine production capacity, and perforin as an indicator of cytotoxic machinery.

Panel 2—Activation and Maturation: Cells were stained with LIVE/DEAD Fixable Blue (Thermo Fisher Scientific), followed by simultaneous surface staining with anti-CD3-BUV496 (BD Biosciences), anti-CD56-PE-Cy7 (BioLegend), anti-CD16-V500 (BD Biosciences), anti-NKG2C-APC (R&D Systems), anti-CD69-BV650 (BD Biosciences), anti-NKG2D-BV785 (BioLegend), and anti-CD57-PerCP-Cy5.5 (BioLegend). The samples were fixed with BD Stabilizing Fixative until acquisition. Of note, these markers were selected to capture distinct aspects of NK cell biology; CD69 as an early activation marker; NKG2D and NKG2C as activating receptors mediating recognition of stressed and virus-infected cells; CD57 as a marker of terminal NK cell differentiation; and NKG2C+CD57+ as the most mature, adaptive-like NK cell phenotype.

Flow cytometry manual data analysis was performed using CytExpert V2.6 and FlowJo software V10. The gating strategy used for analyses is shown in Supplementary Figure S1. Unsupervised analyses were conducted in FlowJo using the UMAP [30], X-Shift [31], and marker enrichment modeling (MEM) [32], plugins for dimensionality reduction, clustering, and cluster characterization, respectively.

### 2.4. Statistical Analysis

All statistical analyses were performed using GraphPad Prism V9 (GraphPad Software). For clinical and demographic comparisons among the three groups, the Kruskal–Wallis test was used, with pairwise comparisons performed using the Mann–Whitney U test; categorical variables were compared using the Chi-square test. For flow cytometry data, the Kruskal–Wallis test with uncorrected Dunn’s post hoc test for comparisons among the three groups was applied. Associations between NK cell subset frequencies and clinical parameters were evaluated using Spearman correlation analysis. A *p*-value < 0.05 was considered statistically significant.

## 3. Results

### 3.1. Clinical Characterization of HCPS Patients

To characterize the clinical and demographic features of our cohort, we analyzed epidemiological and laboratory data from 13 HCPS patients stratified into severe (n = 7) and mild (n = 6) cases, alongside nine healthy donors (Table 1). Within the severe group, early acute phase samples were available only for five or four patients in some flow cytometry panels; the remaining contributed to the convalescent phase samples only. For the mild group, convalescent phase samples were available only for four of six, and acute samples were available for four or six patients depending on flow cytometry panel as well. No significant differences were observed in demographic variables including age, sex, and ethnicity across groups. Briefly, the median age was 29 years (IQR 23–29.5) in severe and 26 years (IQR 21–39) in mild patients, with a predominantly male distribution across both groups (85.7% and 100%, respectively); healthy donors had a median age of 30 years (IQR 28.2–32.2) with a more balanced sex distribution (56% male, 44% female). All the participants were of Amerindian ethnicity. None of the HCPS patients presented relevant comorbidities at the time of enrollment and the healthy donors were individuals in apparent good health, with no chronic diseases and no flu-like symptoms within two weeks prior to enrollment, as specified in the inclusion criteria. As expected, severe patients required a median of 3 days of vasoactive drug support and 3 days of mechanical ventilation, compared to no need in mild patients, while prodromal symptom duration, oxygen support days, hospitalization days, and viral load did not differ significantly between groups. Between the clinical laboratory exams, the platelet counts were below 150 × 10^3^/mm^3^ in all HCPS patients, a hallmark of thrombocytopenia consistently described as an early and surrogate biomarker of hantavirus infection [5]. These clinical and hematological profiles confirm that our cohort stratification accurately reflects the severity spectrum of HCPS. The low incidence of ANDV infection, together with its high case-fatality rate and sporadic geographic distribution, represents a major challenge for the establishment of adequately powered prospective clinical cohorts. Consequently, most published immunological studies of acute hantavirus disease have been conducted in relatively small patient populations. In this context, the cohort analyzed here is comparable in size to those reported in previous investigations of hantavirus immunopathogenesis and represents a valuable and clinically well-characterized cohort for the study of the whole severity spectrum of acute host response to ANDV infection [21,28,33].

### 3.2. CD56dim NK Cell Compartment Is Selectively Depleted and Functionally Impaired in Severe HCPS

#### 3.2.1. Severe HCPS Is Associated with a Shift Toward Less Mature NK Cell Phenotypes

**Figure 1:**
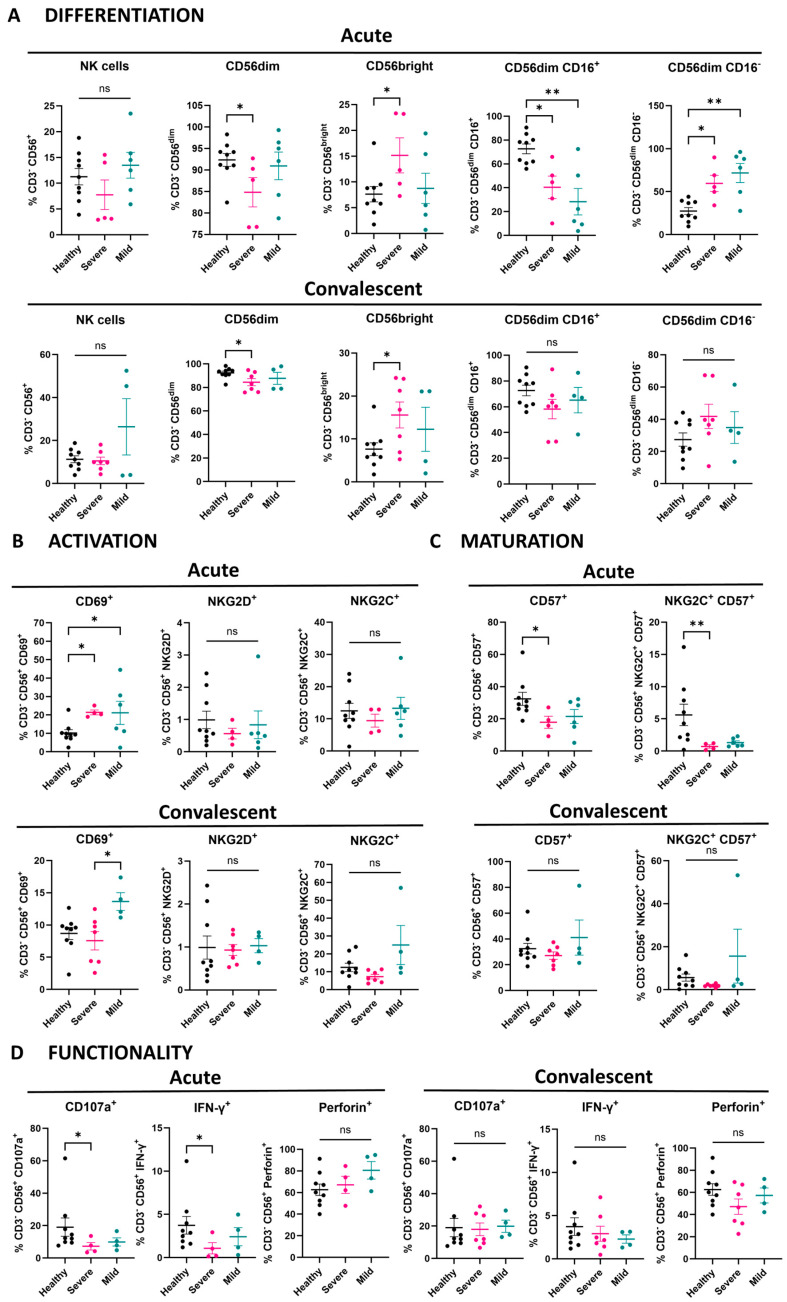
Comprehensive NK cell characterization during acute and convalescent HCPS. (**A**) Differentiation: frequencies of total NK cells (CD3−CD56+), CD56dim, CD56bright, CD56dimCD16+, and CD56dimCD16− subsets. (**B**) Activation: expression of CD69, NKG2D, and NKG2C. (**C**) Maturation: expression of CD57 and NKG2C+CD57+ subset frequency. (**D**) Functionality: expression of CD107a, IFN-γ, and perforin in total K562 stimulated NK cells. The upper panels show the early acute phase (days 0–2) and the lower panels show the convalescent phase (≥60 days). Kruskal–Wallis test with uncorrected Dunn’s test. * *p* < 0.05, ** *p* < 0.01. ns, not significant; NK, natural killer.

To assess whether NK cell differentiation is disrupted during early acute HCPS, we analyzed CD56bright and CD56dim NK cell frequencies, representing early and terminally differentiated stages of NK cell maturation, respectively, alongside their CD16+ and CD16− subpopulations, which further delineate the progression toward full cytotoxic competence, across severity groups and healthy donors, using the gating strategy detailed in Supplementary Figure S1. Total NK cell proportions (CD3−CD56+) did not differ significantly between the clinical outcome during the acute phase (Figure 1A). However, a subset-level analysis revealed striking changes: CD56dim NK cells were significantly reduced in the severe patients compared to the healthy donors; thus, with a reciprocal increase in CD56bright cells in the same group, while the mild patients showed no significant differences in either subset, indicating a strong migration towards infected organs and/or suggesting a selective shift toward a less mature and less cytotoxic NK cell phenotype, specifically in severe clinical outcome. Within the CD56dim phenotype, CD56dimCD16+ NK cells, the most cytotoxically competent circulating NK subset, were significantly reduced in both the mild and severe patients relative to the healthy donors, with an expected increase in CD56dimCD16− cells consequently, across both groups. This pattern indicates a selective loss of the most functionally mature NK cell phenotype during early acute HCPS. This reduction was observed in both mild and severe groups at the CD16 subset level, independent of disease severity. However, the CD56dim/CD56bright ratio was specifically associated with severity, suggesting that redistribution to sites of active viral replication, viral-driven suppression, or both, may underlie this pattern, as has been reported in other viral infections. In the convalescent phase, CD56dim NK cells remained significantly reduced exclusively in the severe patients, with a corresponding persistent increase in CD56bright cells, while all other subsets showed no significant differences, suggesting that severe HCPS leaves a lasting imprint on NK cell subset composition, where the failure to reconstitute the CD56dim phenotype even after clinical recovery suggests a more profound and sustained disruption of NK cell maturation, specifically driven by severe disease (Figure 1A).

#### 3.2.2. Broad NK Cell Activation Across Severity Groups with Divergent Convalescent Responses

To determine whether NK cells are activated during early acute HCPS and whether activation patterns differ by severity, we analyzed CD69, NKG2D, and NKG2C expression across severity groups and healthy donors (Figure 1B). CD69 expression was significantly elevated in both patient groups during the acute phase, compared to the healthy donors, confirming broad NK cell activation across the severity spectrum. Neither NKG2D nor NKG2C showed significant differences at either timepoint, suggesting that receptor-mediated activation pathways are not selectively engaged at this acute phase of infection. In the convalescent phase, elevated CD69 persisted exclusively in the mild patients, potentially reflecting a more sustained and effective antiviral NK cell response in mild disease, a divergence whose functional significance warrants further investigation (Figure 1B).

#### 3.2.3. Terminal NK Cell Differentiation Is Selectively Impaired in Severe HCPS

To evaluate the maturation state of NK cells during early acute HCPS, we analyzed CD57 expression and the NKG2C+CD57+ NK subset across severity groups and healthy donors (Figure 1C). Both CD57 expression and the NKG2C+CD57+ subset were significantly reduced in the severe patients compared to the healthy donors during the acute phase, while the mild patients showed no significant differences. The selective loss of this terminally differentiated phenotype specifically in severe HCPS suggests that the NK cells with the greatest cytotoxic and adaptive-like potential are preferentially depleted or fail to expand during the critical early window of infection, a finding that complements the differentiation data and further supports a severity-specific impairment of NK cell maturation in ANDV infection. Both markers were normalized completely in the convalescent phase across all groups, confirming that this maturation deficit is transient (Figure 1C).

#### 3.2.4. Functional Deficits in Severe HCPS Affect the NK Cells Effector Phenotype

To determine whether the alterations observed in the CD56dim NK cell compartment translate into functional deficits, we analyzed CD107a, IFN-γ, and perforin expression in total NK cells across severity groups and healthy donors under stimulated conditions (Figure 1D). Interestingly, CD107a and IFN-γ were significantly reduced in the severe patients compared to the healthy donors during the acute phase, while the mild patients showed no significant differences; and perforin showed no significant changes across any group or timepoint. All functional markers were normalized in the convalescent phase, confirming that these deficits are transient, and a specific hallmark of HCPS, resolving along with clinical recovery.

### 3.3. Impaired IFN-γ Production and Degranulation Capacity Within CD56dim NK Cells Reveal an Intrinsic Functional Defect in Severe HCPS

#### 3.3.1. CD56dim NK Cells Display Intrinsic IFN-γ Deficiency in Severe HCPS

**Figure 2:**
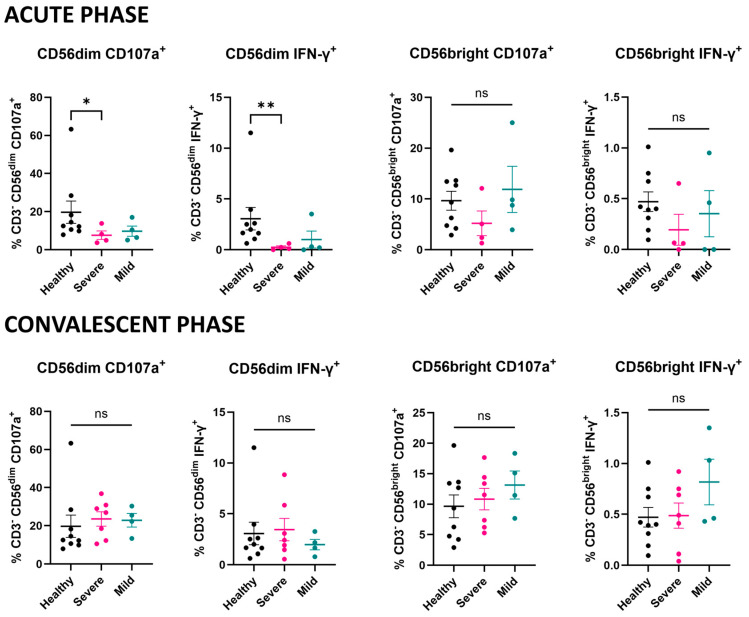
The upper panels show CD107a expression (**left**) and IFN-γ production (**right**) within CD56dim NK cells during the early acute phase (days 0–2). The lower panels show the same markers during the convalescent phase (≥60 days). Kruskal–Wallis test with uncorrected Dunn’s test. * *p* < 0.05, ** *p* < 0.001. ns, not significant.

Given the reduction in CD56dim NK cell numbers observed in the severe patients, we next asked whether the impaired IFN-γ production in whole NK cells reflects a numerical deficit or an intrinsic functional defect within the CD56dim NK remaining cells. To address this, we analyzed IFN-γ production specifically within the CD56dim and CD56bright phenotypes separately, under stimulated conditions, across the severity groups and healthy donors (Figure 2). IFN-γ production was significantly reduced within CD56dim NK cells in the severe patients compared to healthy donors (*p* < 0.01), while the mild patients and CD56bright NK cells showed no significant differences at either timepoint. This finding demonstrates that the IFN-γ deficit in severe HCPS is not merely a consequence of fewer CD56dim cells, instead, it evidences a genuine functional impairment of the remaining CD56dim NK cells, independent of their numerical reduction. This deficit resolved completely in the convalescent phase across all the groups.

#### 3.3.2. Degranulation Capacity Is Intrinsically Impaired Within CD56dim NK Cells in Severe HCPS

To determine whether this functional deficit extends to the subset level, we analyzed CD107a expression within CD56dim and CD56bright NK cells across the severity groups and healthy donors under unstimulated conditions (Figure 2). CD107a expression was significantly reduced within CD56dim NK cells in the severe patients compared to the healthy donors, recapitulating the previous pattern observed for IFN-γ, while the mild patients and CD56bright NK cells remained unaffected at both timepoints. All the groups were normalized in the convalescent phase.

Collectively, the convergent and intrinsic reduction in both IFN-γ production and degranulation capacity within CD56dim NK cells. Notably, an effect independent of their numerical depletion, establishes that severe HCPS is characterized not only by a quantitative reduction in the cytotoxic NK cell compartment but also by a functional defect within CD56dim NK cells. This dual defect positions CD56dim NK cells as the primary locus of NK cell dysfunction in severe ANDV infection, functionally compromised in the acute phase of HCPS, when cytotoxic phenotype is most critical.

### 3.4. Unsupervised Clustering Analysis Independently Validates and Extends the NK Cell Landscape in Severe HCPS

**Figure 3:**
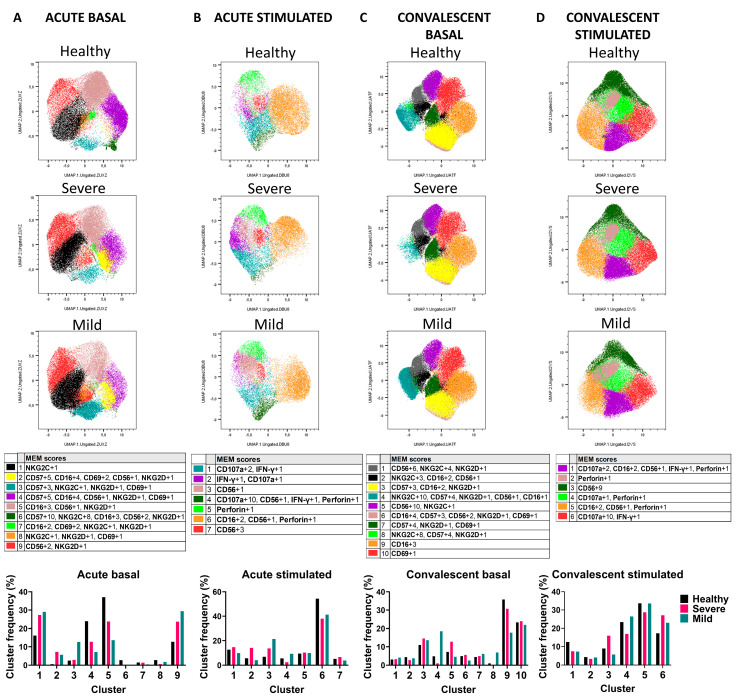
Unsupervised UMAP clustering analysis of NK cell subsets in acute and convalescent HCPS. (**A**) Unstimulated and (**B**) stimulated PBMCs from the healthy donors, severe, and mild HCPS patients during the early acute phase (days 0–2). (**C**) Unstimulated and (**D**) stimulated PBMCs during the convalescent phase (≥60 days). Each UMAP displays cluster distribution across the three groups. Cluster identity was defined by MEM-based annotation of marker enrichment scores. MEM, marker enrichment modeling.

To independently validate our findings through an unbiased approach, we performed unsupervised dimensionality reduction using UMAP combined with X-Shift clustering and MEM-based cluster annotation in unstimulated and stimulated PBMCs from the mild and severe HCPS patients and healthy donors, during both the acute and convalescent phases (Figure 3). During early acute infection, the unsupervised analysis revealed distinct clustering patterns that fully recapitulated the previous gating findings (Figure 3A,B). Clusters 4 and 5, enriched for CD57, CD16, and NKG2D, were markedly reduced in both patient groups, while Cluster 6, with the highest NKG2C and CD57 co-expression, representing the most terminally differentiated adaptive-like phenotype, was nearly absent across both groups, and less differentiated Clusters 1 and 9 expanded reciprocally. In the stimulated panel, a particularly striking functional dichotomy emerged in severe patients: Cluster 4, defined by high co-expression of CD107a, IFN-γ, perforin, and CD56, was reduced to less than half compared to healthy donors (0.44-fold), while Cluster 2, expressing only IFN-γ and CD107a without perforin or CD56, expanded approximately 2.5-fold, suggesting NK cells that are partially activated but unable to acquire full cytotoxic competence, mechanistically extending the intrinsic functional impairment identified in CD56dim NK cells by previous analysis. 

In the convalescent phase, most clusters normalized across the groups, yet unsupervised analysis revealed a divergence not apparent from previous gating alone (Figure 3C,D). Clusters 4 and 8, the two most enriched for NKG2C and CD57 co-expression, showed dramatically divergent reconstitution: while the mild patients expanded both substantially above healthy donor levels (3.8-fold and 7.1-fold, respectively), the severe patients showed a near-complete absence of both (0.20-fold and 0.30-fold). This persistent failure to reconstitute the adaptive-like NK cell pool exclusively in the severe patients, even as functional markers broadly normalized, positions the NKG2C+CD57+ compartment as a potential long-term immunological hallmark of severe ANDV infection.

### 3.5. CD56dimCD16+ NK Cell Frequencies Negatively Correlate with Viral Load During Early Acute HCPS

**Figure 4:**
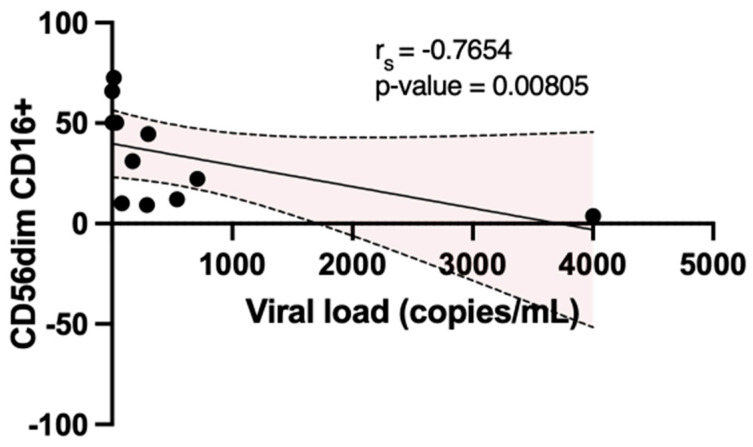
The negative correlation between viral load and CD56dimCD16+ NK cell frequencies in the acute HCPS patients. Spearman correlation analysis revealed a significant negative association between circulating CD56dimCD16+ NK cell proportions and viral load across all the acute HCPS patients (r = −0.765, *p* = 0.008, n = 11). Each dot represents one patient.

To determine whether the reduction in CD56dimCD16+ NK cells is associated with viral load during early acute infection, we performed Spearman correlation analyses between NK cell subset frequencies and a panel of clinical and laboratory parameters across all acute HCPS patients. The CD56dimCD16+ NK cell frequencies correlated negatively and significantly with viral load (r = −0.765, *p* = 0.008, n = 11; Figure 4), independent of clinical severity. No significant associations were found between NK cell subpopulations and any other clinical or laboratory parameter evaluated. The specificity and strength of this association suggest that viral load can influence the CD56dimCD16+ NK cell phenotype during the earliest phase of HCPS, raising the question of whether higher viral loads directly suppress this population, whether its depletion permits greater viral replication, or whether higher viral loads drive cytokine production that in turn modulates this NK cell subset; and positions CD56dimCD16+ cells as a potential early immunological indicator of innate antiviral control in ANDV infection.

## 4. Discussion

In this study, we characterized the NK cell landscape during early acute HCPS caused by ANDV in Chilean patients stratified by clinical severity. Our findings reveal a coordinated, severity-specific disruption of the CD56dim NK cell compartment, affected simultaneously at the level of differentiation, maturation, and intrinsic effector function, while total NK cell proportions remained unaffected. Consistent with observations in other severe viral infections and sepsis [34], CD56dimCD16+ NK cells, one of the most cytotoxically competent NK phenotypes, were reduced across both mild and severe patients compared to the healthy donors, while additional deficits in terminal maturation and cytotoxic function were evidenced exclusively in the severe patients and persisted in part beyond clinical recovery. Furthermore, CD56dimCD16+ NK cell frequencies correlated negatively and significantly with viral load across all the acute HCPS patients, independent of clinical severity, positioning viral load as an active modulator of the most functionally competent NK subset during the earliest phase of infection, and raising the question of whether this depletion reflects active viral suppression, redistribution to sites of infection, or both.

The selective reduction in CD56dim NK cells during severe systemic viral infection is a pattern increasingly recognized across distinct pathological contexts. For example, in sepsis, the depletion of circulating NK cells, particularly the CD56dim subset, correlates with disease severity and poor clinical outcomes [26,34], with impaired IFN-γ production observed specifically in patients with septic shock [35]. In acute COVID-19, NK cells are rapidly mobilized from peripheral blood toward the lungs, a redistribution pattern explicitly noted to parallel what is observed in acute hantavirus infection as well from this work and others [26,28], with impaired NK cell counts and cytolytic activity characterizing severe disease [36,37]. Notably, in sepsis this peripheral NK cell depletion has been attributed primarily to apoptosis rather than tissue migration, as NK cell levels in bronchoalveolar lavage remain largely unchanged despite marked peripheral lymphopenia [38]. This distinction is particularly relevant in the context of our findings: the absence of significant changes in total NK cell proportions in HCPS patients, alongside the selective and phenotype-specific depletion of CD56dim and CD56dimCD16+ subsets, suggests that the observed loss is not simply a consequence of redistribution to infected tissues; instead, it suggests an active and selective process, potentially involving apoptosis and/or viral-driven suppression, that affects the most functionally mature NK cell phenotypes. What further distinguishes our findings is not the quantitative reduction alone, but the convergent failure across differentiation, maturation, and functionality, specifically to the CD56dim phenotype in severe HCPS, positioning the HCPS not merely as a state of NK cell depletion, but as one of specific functional impairment of the most cytotoxically competent NK cell population at the precise moment when innate antiviral control is most critical.

The reciprocal increase in CD56bright NK cells observed in severe patients should not be interpreted as a compensatory response, rather, it reflects the residual pool that persists when the most cytotoxically competent cells are lost. CD56bright NK cells, while capable of cytokine production, lack the full cytotoxic machinery of their CD56dim counterparts and cannot restore effector capacity, including CD16-mediated ADCC, a mechanism shown to correlate with reduced disease severity in acute influenza virus infection [39,40] and likely critical for the containment of ANDV-infected endothelium [21,26].

Despite the functional deficits observed in the severe patients, CD69 expression was significantly elevated in both groups during the acute phase, consistent with the cytokine-driven, receptor-independent NK cell activation characteristic of early acute viral infection [26], and previously described in both hantavirus and COVID-19 contexts [21,37]. The absence of significant NKG2D and NKG2C changes further supports a non-selective activation pattern at this early timepoint. Notably, CD69 elevation persisted exclusively in the mild patients during convalescence, a divergence that may reflect either a more sustained antiviral response in mild disease, or the emergence of a tissue-resident NK cell phenotype, given the established role of CD69 as a canonical tissue-residency marker [26]. A similar persistence of NK cell activation markers during convalescence has been described in mild COVID-19 and PUUV-driven HFRS [21,37], suggesting that sustained NK cell engagement may be a shared feature of effective viral control across distinct hemorrhagic and respiratory viral infections, in contrast to the resolution pattern observed in severe disease.

The reduction in CD57 expression and the NKG2C+CD57+ adaptive-like NK cell subset exclusively in the severe patients adds a mechanistically significant dimension to these findings. Beyond their role as markers of terminal differentiation, NKG2C+CD57+ NK cells undergo long lasting epigenetic remodeling at the IFNG locus, specifically demethylation of the conserved non-coding sequence 1 (CNS1), conferring constitutive chromatin accessibility and enhanced IFN-γ production capacity upon viral stimulation [41,42]. This specific depletion in severe HCPS therefore implies not only the loss of terminally differentiated cytotoxic cells, but also the elimination of the NK cell pool with a prime state to antiviral cytokine production, a deficit that likely contributes directly to the impaired IFN-γ output observed in the CD56dim NK cells of the severe patients. These observations are in contrast to severe COVID-19, where adaptive-like NKG2C+CD57+ NK cells expand rather than contract [43], suggesting that ANDV may actively suppress this subset, or that severe HCPS patients lack the CMV-primed pool required for its expansion, a distinction that contributes to delineate for differences between these two viral diseases.

In our study, the NK cells from the severe patients displayed reduced CD107a expression and IFN-γ production following K562 stimulation, indicating impaired effector function, whereas intracellular perforin levels remained unchanged. This observation is consistent with previous studies showing that K562 stimulation does not necessarily induce a measurable decrease in intracellular perforin as detected by flow cytometry [44,45]. Indeed, CD107a has been highlighted as a more sensitive and reliable marker of NK cell degranulation than intracellular perforin, as it directly reflects lytic granule exocytosis [46,47], whereas intracellular perforin primarily represents the total cytolytic granule content rather than its release. The unchanged perforin levels observed in our study are therefore consistent with impaired NK cell effector function in severe HCPS.

The convergent reduction in both IFN-γ production and CD107a-mediated degranulation within the CD56dim NK cells of HCPS severe patients, independent of their reduction, establishes that severe HCPS is characterized not only by a NK cell loss, but also by a functional impairment of the cells that remain. This impairment between activation, evidenced by elevated CD69, and effector function markers decreased as the impaired IFN-γ and CD107a, specifically in CD56dim cells, mirrors the misdirected and exhausted NK cell response described in severe COVID-19 [26], and is further supported by unsupervised clustering analysis: in the stimulated panel, the cluster most enriched for cytotoxic markers, defined by the high co-expression of CD107a, IFN-γ, perforin, and CD56, also was reduced to less than half its frequency in the severe patients (0.44-fold), while a distinct cluster expressing only IFN-γ and CD107a without perforin expanded approximately 2.5-fold. Notably, the absence of intracellular perforin in this CD107a+ cluster is consistent with recent degranulation, as surface CD107a translocation is a well-established marker of granule exocytosis. Future studies characterizing the cytotoxic versus cytokine-producing capacity of this population in the context of ANDV infection will help further define its functional role. These findings provide unbiased evidence of NK cells that are activated but blocked from acquiring complete effector competence. Whether this functional arrest reflects inhibition of granzyme B and caspase-3 signaling [21], activation-induced exhaustion, or the cumulative inflammatory environment of early HCPS, remains an open question.

Some limitations of this study are worth acknowledging. Although the sample size is modest, it represents a meaningful cohort given the rarity and lethality of HCPS, providing a robust basis for identifying immunological patterns and generating hypotheses for future investigation. The samples were collected at days 0–2 of cardiopulmonary symptom onset, the earliest clinically accessible window in HCPS, making this study one of the first characterizations of NK cell dynamics at disease onset; notably, this early sampling window also means that only two of the five severe patients had received vasoactive drugs in close proximity to sample collection. Although these agents may exert some degree of NK cell immunomodulation [48], total NK cell proportions remained unchanged and perforin expression was unaffected, and the observed alterations were consistent across all the severe patients regardless of vasoactive drug exposure, arguing against a predominant pharmacological confound. Nevertheless, longitudinal sampling across the full acute phase would further enrich the immunological state described here. Furthermore, unlike HFRS caused by Old World hantaviruses, which accounts for an estimated 150,000 cases annually in Eurasia, ANDV-driven HCPS is endemic of South America with only 40–60 confirmed cases per year in Chile. Notably, even in regions with substantially higher hantavirus prevalence, landmark immunological studies of NK cell responses during acute hantavirus disease have been conducted with comparable cohort sizes [21,28,33], underscoring that these sample sizes reflect the inherent constraints of studying rare and rapidly progressive infections rather than a limitation specific to this study.

## 5. Conclusions

In this study, we provide the first comprehensive characterization of the NK cell landscape during early acute HCPS caused by ANDV, revealing a coordinated, severity-specific disruption of the CD56dim NK cell compartment at the level of differentiation, maturation, and intrinsic effector function. These findings establish that severe HCPS is not merely a state of NK cell depletion, but one of selective functional impairment of the most cytotoxically competent NK cell population at the critical early phase of infection. The negative correlation between CD56dimCD16+ NK cell frequencies and viral load further supports an active role of viral dynamics in shaping innate immune competence during early HCPS. The intrinsic deficits identified in CD56dim NK cells position this population as a potential therapeutic target, where strategies aimed at restoring NK cell effector function may help limit disease progression.

## Figures and Tables

**Table 1:** Demographics and clinical characteristics of HCPS patients by severity.

	Severe	Mild	Healthy Donors	*p* Value
No. of patients	7	6	9	
Age (years),	29 (23.0–29.5)	26 (21.0–39.0)	30 (28.2–32.2)	1.000 ^a^
Median (25 to 75% IQR)				
Gender, n (%)				
Male	6 (85.7)	6 (100)	5 (56)	0.125 ^b^
Female	1 (14.3)	0	4 (44)	
Ethnicity, n (%)				
European	0	0	0	NA
Amerindian	7	6	9	
Days with prodromal symptoms, median	4 (3.8–8.0)	4 (3.0–5.0)	NA	1.000 ^c^
(25 to 75% IQR)				
Days in oxygen	6 (6.0–7.5)	3 (2.0–7.0)	NA	0.643 ^c^
Days in VD	3 (3.0–5.2)	0	NA	0.000 ^c^
Days in MV	3 (2.8–5.2)	0	NA	0.000 ^c^
Days in ECMO	0	0	NA	NA
Hospitalization Days	6 (6.0–7.5)	3 (2.0–7.0)	NA	0.533 ^c^
Laboratory exams ^d^				
Viral load	83 (5.4–170)	415 (98–668)	NA	1.000 ^c^
(copies/mL)				
Platelets (cells/mm^3^)	82,000 (70,000–131,250)	79,000 (51,000–89,000)	NA	1.000 ^c^
Leucocytes (cells/mm^3^)	10,150 (6600–19,675)	9200 (6700–10,250)	NA	0.700 ^c^
Neutrophils %	54.7 (54.4–58.5)	60.5 (58.0–62.3)	NA	1.000 ^c^

Abbreviations: HCPS, hantavirus cardiopulmonary syndrome; NA, not applicable; VD, vasoactive drugs; MV, mechanical ventilation; ECMO, extracorporeal membrane oxygenation; n, number. “^a^” The Kruskal–Wallis test was used for comparisons between the severe case group, the mild case group and the healthy controls. “^b^” The Chi-square test was used for comparisons of categorical variables (sex, ethnicity) between groups. “^c^” The Mann–Whitney test was used for comparisons between the severe and mild case groups. “^d^” The data shown represents the median (25 to 75% IQR) of the first available sample of each patient (days 0–2 post-cardiopulmonary symptom onset).

## Data Availability

The original contributions presented in this study are included in the article/Supplementary Material. Further inquiries can be directed to the corresponding author.

## Supplementary Materials

The following supporting information can be downloaded at: https://www.mdpi.com/article/10.3390/v18070712/s1. Figure S1: Flow cytometry gating strategy for NK cell phenotyping and functional analysis. NK cells were identified as CD3^−^CD56^+^ cells and further subdivided into CD56bright and CD56dim populations, including CD56dim CD16^+^ and CD56dim CD16^−^ subsets. Phenotypic analysis included the evaluation of CD57, CD69, NKG2C, NKG2D and NKG2C^+^CD57^+^ expression. Functional analysis after stimulation included the assessment of perforin, CD107a, and IFN-γ expression within NK cells and CD56dim and CD56bright subsets.

## Abbreviations

The following abbreviations are used in this manuscript:

ANDV	Orthohantavirus andesense
ADCC	Antibody-dependent cellular cytotoxicity
ARDS	Acute respiratory distress syndrome
CD	Cluster of differentiation
CMV	Cytomegalovirus
CNS1	Conserved non-coding sequence 1
CXCL10	C-X-C motif chemokine ligand 10
ECMO	Extracorporeal membrane oxygenation
HCPS	Hantavirus cardiopulmonary syndrome
HD	Healthy donors
HFRS	Hemorrhagic fever with renal syndrome
IFN-γ	Interferon-gamma
IL	Interleukin
MEM	Marker enrichment modeling
MV	Mechanical ventilation
NK	Natural killer
NKG2C	NK group 2 member C
NKG2D	NK group 2 member D
PBMC	Peripheral blood mononuclear cell
PUUV	Puumala orthohantavirus
RdRp	RNA-dependent RNA polymerase
RT-qPCR	Reverse transcription quantitative polymerase chain reaction
UMAP	Uniform manifold approximation and projection
VD	Vasoactive drugs
