# Supplementary material for: The Natural Killer Cell Landscape in the Natural History of Hantavirus Cardiopulmonary Syndrome in a Chilean Cohort
Source: Viruses. 2026 Jun 27;18(7):712. doi: 10.3390/v18070712 (PMC13431568; doi:10.3390/v18070712)
Supplement: Supplementary file 1 [file viruses-18-00712-s001.zip › viruses-4373506-supplementary.pdf]

SUPPLEMENTARY FIGURE

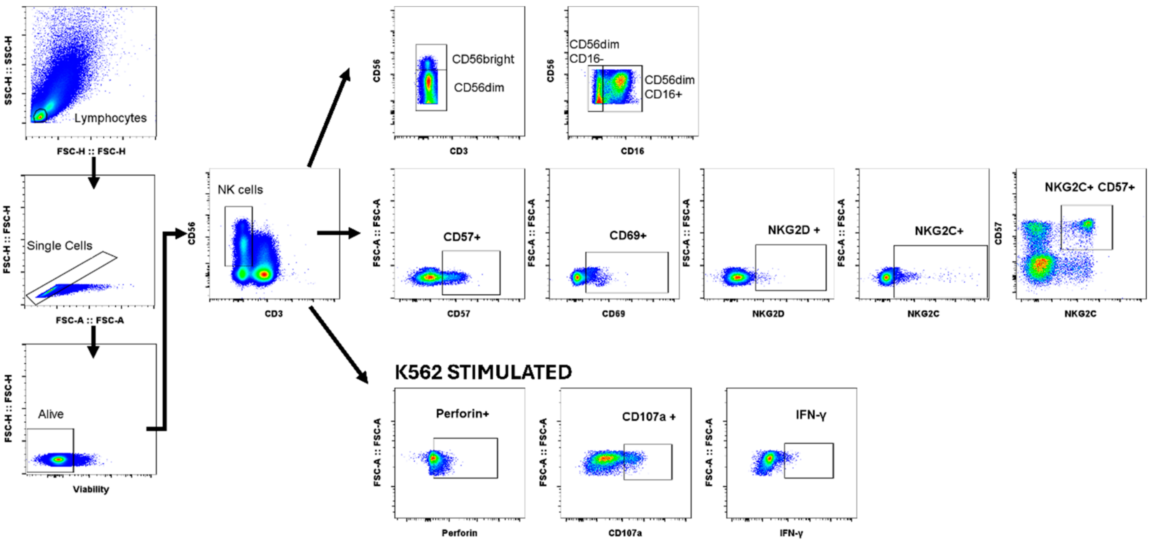

Supplementary Figure S1: Flow cytometry gating strategy for NK cell phenotyping and functional analysis. NK cells were identified as $CD3^{-}CD56^{+}$ cells and further subdivided into CD56bright and CD56dim populations, including CD56dim $CD16^{+}$ and CD56dim $CD16^{-}$ subsets. Phenotypic analysis included the evaluation of CD57, CD69, NKG2C, NKG2D and $NKG2C^{+}CD57^{+}$ expression. Functional analysis after stimulation included the assessment of perforin, CD107a, and IFN-γ expression within NK cells and CD56dim and CD56bright subsets.
